# Prevalence of All-Cause and Diagnosis-Specific Disability Pension at the Time of First Coronary Revascularisation: A Population-Based Swedish Cross-Sectional Study

**DOI:** 10.1371/journal.pone.0115540

**Published:** 2015-01-28

**Authors:** Katharina Zetterström, Margaretha Voss, Kristina Alexanderson, Torbjörn Ivert, Kenneth Pehrsson, Niklas Hammar, Marjan Vaez

**Affiliations:** 1 Division of Insurance Medicine, Department of Clinical Neuroscience, Karolinska Institutet, Stockholm, Sweden; 2 Department of Analysis and Forecasts, Swedish Social Insurance Agency, Stockholm, Sweden; 3 Department of Cardiothoracic Surgery and Anesthesiology, Karolinska University Hospital and Department of Molecular Medicine and Surgery, Karolinska Institutet, Stockholm, Sweden; 4 Department of Medicine, Karolinska Institutet, Stockholm, Sweden; 5 AstraZeneca R&D, Mölndal, Sweden; 6 Institute of Environmental Medicine, Karolinska Institutet, Stockholm, Sweden; 7 Centre for Occupational and Environmental Medicine, Stockholm County Council, Stockholm, Sweden; University of Bologna, ITALY

## Abstract

**Background:**

Although coronary revascularisation by coronary artery bypass graft surgery (CABG) and percutaneous coronary intervention (PCI) is well documented, scientific knowledge on disability pension (DP) at the time of revascularisation is lacking. The aim was to investigate the prevalence of all-cause and diagnosis-specific DP at the time of a first coronary revascularisation, accounting for socio-demographic and medical factors.

**Materials and Methods:**

A population-based cross-sectional study using Swedish registers was conducted including all 65,676 patients (80% men) who when aged 30–63 years, within 1994–2006, had a first CABG (n = 22,959) or PCI (n = 42,717) and did not have old-age pension. Associations between socio-demographic and medical factors and the probability of DP were estimated by odds ratios (OR) with 95% confidence intervals (CI) using logistic regression analyses.

**Findings:**

The prevalence of DP at time of revascularisation was 24%, mainly due to musculoskeletal diagnoses. Sixty-two percent had had DP for at least four years before the revascularisation. In the multivariable analyses, DP was more common in women (OR: 2.40; 95% CI: 2.29–2.50), older patients (50–63 years); especially men aged 60–63 years with CABG (OR: 4.91; 95% CI: 4.27–5.66), lower educational level; especially men with PCI (OR: 2.96; 95% CI: 2.69–3.26), patients born outside Sweden; especially men with PCI (OR: 2.11; 95% CI: 1.96–2.27), and in women with an indication of other diagnoses than acute coronary syndrome (ACS) or stable angina pectoris for PCI (OR: 1.72; 95% CI: 1.31–2.24).

**Conclusion:**

About a quarter had DP at the time of revascularisation, often due to musculoskeletal diagnoses. More than half had had DP for at least four years before the intervention. DP was associated with female gender, older age, lower educational level, and being born outside Sweden.

## Introduction

Annually in Sweden about 10,000 patients of working age undergo coronary revascularisation, i.e. coronary artery bypass graft surgery (CABG) or percutaneous coronary intervention (PCI). These are established and well-documented interventions [1–8] resulting in symptom reduction, improved physical capacity, and reduced mortality among patients with ischemic heart disease, including acute coronary syndrome (ACS) and stable angina pectoris [6]. Coronary revascularisation could hence increase the possibility of return to work [9]. And alertness is warranted in health care, regarding individualised rehabilitation measures, to promote return to work. Nevertheless, some patients might already be on disability pension (DP) at the time of revascularisation and thus, already permanently excluded from the work force. Also, over the last decades, DP has increased in many western countries [10,11]. However, no studies have, so far, investigated the extent of DP at the time of coronary revascularisation – and even less is known about the diagnoses for DP in this patient group. Such knowledge is of importance when planning for what type of rehabilitation measures to offer patients with coronary revascularisation. In general, DP is more common among women [12–22], older individuals [23,24], lower level educated [17,25–29] and foreign-born individuals [30–33]. Whether this also applies for patients undergoing coronary revascularisation is not scientifically known. Results from studies on DP rates could be attrition biased by drop outs, and the generalisability of findings could be affected when only a few clinics are included. Also employment frequency might affect results – if those older than 55 or 60 years are not gainfully employed, and if women are less employed than men, this would imply an age- or gender bias in who applies for DP. As Sweden has one of the highest employment frequencies, also regarding people of higher age and of female gender [34,35], it would be an advantage to base such a study on all coronary revascularisations conducted in Sweden. The aim of this study was to investigate the prevalence of all-cause and diagnosis-specific DP at the time of a first coronary revascularisation, accounting for socio-demographic and medical factors.

## Materials and Methods

### Ethics Statement

The study population was identified through nationwide registers collected and stored with the consent of the patients. Additional information was collected by linkage of several public national registers. Ethical vetting is always required when using register data in purpose of research in Sweden. The ethical vetting is performed by regional ethical review boards and the risk appraisal associated with the Law on Public Disclosure and Secrecy is done by data owners. The ethical review boards can however waive the requirement to consult the data subjects directly to obtain their informed consent, and will often do so if the research is supported by the ethical review board and the data has already been collected in some other context. According to these standards in Sweden this project has been evaluated and approved by the Regional Ethical Review Board of Stockholm, Sweden (2006/661-31).

### Study population

This population-based register study comprised all the 65,676 (80% men) individuals in Sweden, who within 1994–2006, when aged 30–63 years, had a first CABG (n = 22,959) or PCI (n = 42,717) and did not have old-age pension. The patients were identified using the nationwide quality register for coronary revascularisation SWEDEHEART [3] including information on patient characteristics, date and type of intervention for all performed coronary revascularisations in Sweden.

### Linkage to Nationwide Registers

The information from nationwide registers was linked to each patient using the unique Swedish personal identification number. Information on date, degree and diagnosis of DP was obtained from the Swedish Social Insurance Agency (MiDAS data base). Information on level of education, country of birth, and type of living area was obtained from the Statistics Sweden (LISA register). And, if data was not available in SWEDEHEART, information on diabetes mellitus and indication for intervention was obtained from the Board of Health and Welfare (The National Patient Register).

### Outcome

The outcome was having DP at the time of a first CABG or PCI.

### Categorisation of variables

DP diagnoses were classified according to International Classification of Diseases version 10 (ICD-10) [36] and categorised into five groups: Cardiovascular diseases (I00-99) (referred as ‘CVD’), mental and behavioural disorders (F00-F99) (‘mental diagnoses’), diseases of the musculoskeletal system and connective tissue (M00-M99) (‘musculoskeletal diagnoses’), all ‘other diagnoses’, and ‘no information’. Information about DP diagnoses was not available for most of the DPs granted before the year 1994. Degree of DP was categorised as part- (≤50%) and full-time DP (>50%). Years on DP before intervention were categorised into ≤3, 4–10, and 11–35 years. Socio-demographic and medical factors were categorised as follows. Age at intervention: 30–49, 50–54, 55–59, and 60–63 years. Level of education: elementary school (≤9 years), high school (>9 and ≤12 years), and college/university (>12 years). Country of birth: Sweden and other countries. Type of living area: larger cities (Stockholm, Gothenburg, Malmö), medium- sized cities, and smaller communities [37]. Year of intervention: 1994–1996, 1997–2000, 2001–2003, and 2004–2006. Indication for intervention was categorised according to the ICD-10 into: acute coronary syndrome (ACS) with non-ST-elevated myocardial infarction or rarely ST-elevated myocardial infarction, stable angina, and others. Diabetes mellitus: yes, no and missing data (12%). A sensitivity analysis of the missing diabetes data revealed no gender or age differences between the missing and the obtained data. However, a somewhat higher proportion of lower educational level and CABG was seen in patients with missing data.

### Statistical analyses

Descriptive statistics were used to outline study-population characteristics and prevalence of all-cause and diagnosis-specific DP at the time of coronary revascularisation. Crude and adjusted odds ratios (OR) with 95% confidence interval (CI) for DP at the time of revascularisation were calculated by logistic regression analyses. Significant covariates from the univariable model were included in the multivariable analyses. The ORs were adjusted for covariates in the following models; model I (age), model II (age and level of education), model III (gender), and model IV (all variables included). Most analyses were stratified by gender and type of intervention (CABG, PCI).

### Social insurance in Sweden

In Sweden, all individuals aged 19–64 years with long-term or permanent work incapacity due to disease or injury can be granted DP for part- or full-time of ordinary working hours. The common age for old-age pension is 65, but it can be obtained earlier. For individuals with no or a low previous income, the DP benefits amount to a minimum level. For those with a previous income, the benefits amount to at least 64% of lost income and up to a certain level.

## Results

The characteristics of the study population and the prevalence of DP stratified by type of intervention and gender are presented in Table 1. Mean age at intervention was 55 years and a majority had at most a high school education, were born in Sweden, and lived in medium-sized cities and smaller communities. Most had their first PCI during year 2004–2006 whereas most CABG procedures were performed before 2001. ACS was the main indication for revascularisation in a majority of the patients (61%). Eighteen percent had diabetes mellitus at the time of revascularisation. The prevalence of DP at the time of revascularisation was 24% and the highest prevalence was found among women with CABG (41%)

**Table 1:** Patient characteristics, stratified by type of intervention and gender, of all patients in Sweden (N = 65,676), 30–63 years of age, with a first coronary artery bypass graft surgery (CABG) or percutaneous coronary intervention (PCI) within the year 1994–2006, and with disability pension (DP) (n = 15,497) at time of intervention.

	**CABG (n = 22,959)**				**PCI ( n = 42,717)**			
	**Women**		**Men**		**Women**		**Men**	
	**All (n)**	**DP%^1^**	**All (n)**	**DP%^1^**	**All(n)**	**DP%^1^**	**All (n)**	**DP%^1^**
**All**	3671	40.9	19,288	23.1	9384	36.3	33,333	18.4
**Age at intervention (years)**								
30–49	518	24.5	2582	10.8	1705	22.6	6887	9.8
50–54	658	32.8	3981	15.2	1939	28.3	7443	13.5
55–59	1160	40.9	6548	21.6	2901	37.2	10759	19.2
60–63	1335	51.2	6177	35.0	2839	48.9	8244	29.1
**Level of education**								
College or university	485	26.0	3290	12.5	1471	22.3	6625	9.4
High school	1595	36.4	7964	21.6	4404	34.7	14612	17.7
Elementary school	1549	50.5	7890	29.0	3413	44.7	11883	24.2
Missing data	42	23.8	141	27.7	96	22.9	213	31.0
**Country of birth**								
Sweden	2968	39.1	15790	21.3	7753	34.6	27202	16.7
Other	703	48.3	3498	31.2	1631	44.5	6131	26.1
**Type of living area (cities and communities)**								
Larger	1035	35.7	6049	21.1	2746	33.0	10135	18.2
Medium	1281	40.6	6698	22.6	3582	37.4	12541	17.8
Smaller	1272	44.6	6238	24.5	2889	37.3	10147	18.7
Missing data	83	51.8	303	44.2	167	47.3	510	30.8
**Year of intervention**								
1994–1996	1020	42.3	5570	25.9	1189	34.8	4243	20.4
1997–2000	1287	39.4	6372	23.2	2257	33.3	7536	18.0
2001–2003	807	40.1	4300	20.3	2733	34.9	9699	17.4
2004–2006	557	42.7	3041	21.8	3205	40.1	11855	18.7
**Indication for intervention**								
ACS^2^	2063	42.0	10625	24.4	5895	35.8	21133	18.1
Stable angina pectoris	1414	39.4	7605	21.2	3207	36.5	11132	18.9
Other	139	42.4	646	26.9	279	44.4	1039	20.0
Missing data	55	30.9	412	18.7	3	66.7	29	37.9
**Diabetes mellitus**								
No	2093	35.3	12645	19.9	6560	33.1	24749	16.4
Yes	1132	52.6	3871	33.1	1823	50.6	5021	28.2
Missing data	446	37.2	2772	23.8	1001	30.8	3563	18.8

^1^ Proportion of patients with disability pension at the time of first coronary revascularisation

^2^ Acute Coronary Syndrome: Non-ST-elevated myocardial infarction or ST-elevated myocardial infarction

Descriptive data for all-cause and diagnosis-specific DP is presented in Table 2. The largest DP-diagnostic group was musculoskeletal diagnoses (28% of all; and 41% of all with information available on DP diagnoses). This was also the largest diagnostic group in most subgroups. The second largest DP-diagnostic group was CVD (15%; 22% among those with diagnoses). Among those with DP diagnoses, DP due to CVD was most common in men with revascularisation before year 1996. The third largest DP-diagnostic group was mental diagnoses (9%; 13% among those with diagnoses). Musculoskeletal and mental DP diagnoses were about three times more common among the PCI patients compared to CABG. Of all on DP at the time of revascularisation, 89% had full-time DP and 43% were 60–63 years of age. Sixty-two percent had had DP for at least four years before the revascularisation.

**Table 2:** Frequencies (n) and proportions (%) of disability pension (DP) diagnoses among women and men with a first coronary revascularisation within the year 1994–2006 (n = 15,497).

	**All with DP at intervention**		**CVD (I00-99)^1^**		**Mental (F00-99)^1^**		**Musculoskeletal (M00-99)^1^**		**Other^1^**		**No information**	
	**Women**	**Men**	**Women**	**Men**	**Women**	**Men**	**Women**	**Men**	**Women**	**Men**	**Women**	**Men**
	**n (%)^2^**	**n (%)^2^**	**n (%)^3^**	**n (%)^3^**	**n (%)^3^**	**n (%)^3^**	**n (%)^3^**	**n (%)^3^**	**n (%)^3^**	**n (%)^3^**	**n (%)^3^**	**n (%)^3^**
	4,903 (31.6)	10,594 (68.4)	502 (10.2)	1,796 (17.0)	397 (8.1)	947 (8.9)	1,568 (32.0)	2,763 (26.1)	824 (16.8)	1,809 (17.1)	1,612 (32.9)	3,279 (31.0)
**Type of intervention**												
CABG	1500 (30.6)	4458 (42.1)	188 (12.5)	850 (19.1)	73 (4.9)	269 (6.0)	349 (23.3)	976 (21.9)	258 (17.2)	678 (15.2)	632 (42.1)	1685 (37.8)
PCI	3403 (69.4)	6136 (57.9)	314 (9.2)	946 (15.4)	324 (9.5)	678 (11.0)	1219 (35.8)	1787 (29.1)	566 (16.6)	1131 (18.4)	980 (28.8)	1594 (26.0)
**Age at intervention (years)**												
30–49	513 (10.5)	951 (9.0)	50 (9.7)	110 (11.6)	76 (14.8)	193 (20.3)	167 (32.6)	235 (24.7)	134 (26.1)	244 (25.7)	86 (16.8)	169 (17.8)
50–54	764 (15.6)	1607 (15.2)	80 (10.5)	287 (17.9)	80 (10.5)	232 (14.4)	253 (33.1)	422 (26.3)	149 (19.5)	298 (18.5)	202 (26.4)	368 (22.9)
55–59	1554 (31.7)	3476 (32.8)	170 (10.9)	636 (18.3)	109 (7.0)	297 (8.5)	536 (34.5)	953 (27.4)	278 (17.9)	568 (16.3)	461 (29.7)	1022 (29.4)
60–63	2072 (42.3)	4560 (43.0)	202 (9.7)	763 (16.7)	132 (6.4)	225 (4.9)	612 (29.5)	1153 (25.3)	263 (12.7)	699 (15.3)	863 (41.7)	1720 (37.7)
**Level of education**												
College or university	454 (9.3)	1031 (9.7)	61 (13.4)	233 (22.6)	58 (12.8)	177 (17.2)	130 (28.6)	181 (17.6)	139 (30.6)	232 (22.5)	66 (14.5)	208 (20.2)
High school	2109 (43.0)	4298 (40.6)	227 (10.8)	760 (17.7)	207 (9.8)	448 (10.4)	767 (36.4)	1209 (28.1)	403 (19.1)	803 (18.7)	505 (23.9)	1078 (25.1)
Elementary school	2308 (47.1)	5160 (48.7)	213 (9.2)	788 (15.3)	125 (5.4)	307 (5.9)	665 (28.8)	1348 (26.1)	273 (11.8)	766 (14.8)	1032 (44.7)	1951 (3.8)
**Country of birth**												
Sweden	3842 (78.4)	7906 (74.6)	418 (10.9)	1387 (17.5)	317 (8.3)	632 (8.0)	1240 (32.3)	2091 (26.4)	681 (17.7)	1456 (18.4)	1186 (30.9)	2340 (29.6)
Other	1061 (21.6)	2686 (25.4)	84 (7.9)	407 (15.2)	80 (7.5)	315 (11.7)	328 (30.9)	672 (25.0)	143 (13.5)	353 (13.1)	426 (40.2)	939 (35.0)
**Type of living area (cities and communities)**												
Larger	1275 (26.0)	3124 (29.5)	140 (11.0)	514 (16.5)	146 (11.5)	404 (12.9)	352 (27.6)	711 (22.8)	249 (19.5)	564 (18.1)	388 (30.4)	931 (29.8)
Medium	1861 (38.0)	3750 (35.4)	172 (9.2)	603 (16.1)	150 (8.1)	305 (8.1)	631 (33.9)	1043 (27.8)	276 (14.8)	614 (16.4)	632 (34.0)	1185 (31.6)
Smaller	1645 (33.6)	3429 (32.4)	178 (10.8)	623 (18.2)	94 (5.7)	222 (3.5)	554 (33.7)	971 (28.3)	275 (16.7)	569 (17.4)	544 (33.1)	1044 (30.4)
**Indication for intervention**												
Stable angina	1726 (35.2)	3715 (35.1)	216 (12.5)	751 (20.2)	116 (6.7)	255 (6.9)	543 (31.5)	951 (25.6)	285 (16.5)	653 (17.6)	566 (32.8)	1105 (29.7)
ACS^4^	2975 (60.7)	6409 (60.5)	259 (8.7)	960 (15.0)	262 (8.8)	662 (10.3)	985 (33.1)	1717 (26.8)	491 (16.5)	1054 (16.4)	978 (32.9)	2016 (31.5)
Other	183 (3.7)	382 (3.6)	25 (13.7)	68 (17.8)	18 (9.8)	25 (6.5)	37 (20.2)	76 (19.9)	46 (25.1)	92 (24.1)	57 (31.1)	121 (31.7)
**Year of intervention**												
1994–1996	845 (17.2)	2306 (21.8)	98 (11.6)	404 (17.5)	9 (1.1)	49 (2.1)	125 (14.8)	287 (12.4)	61 (7.2)	192 (8.3)	552 (65.3)	1374 (59.6)
1997–2000	1258 (25.7)	2839 (26.8)	156 (12.4)	576 (20.3)	59 (4.7)	164 (5.8)	329 (26.2)	678 (23.9)	192 (15.3)	442 (15.6)	522 (41.5)	979 (34.5)
2001–2003	1278 (26.1)	2565 (24.2)	129 (10.1)	436 (17.0)	115 (9.0)	279 (10.9)	465 (36.4)	825 (32.2)	245 (19.2)	507 (19.8)	324 (25.4)	518 (20.2)
2004–2006	1522 (31.0)	2884 (27.2)	119 (7.8)	380 (13.2)	214 (14.1)	455 (15.8)	649 (42.6)	973 (33.7)	326 (21.4)	668 (23.2)	214 (14.1)	408 (14.1)
**Degree of DP**												
>50% DP	4375 (89.2)	9391 (88.6)	451 (10.3)	1564 (16.7)	366 (8.4)	875 (9.3)	1360 (31.1)	2397 (25.5)	724 (16.5)	1566 (16.7)	1474 (33.7)	2989 (31.8)
≤50% DP	516 (10.5)	1186 (11.2)	51 (9.9)	230 (19.4)	31 (6.0)	72 (6.1)	207 (40.1)	366 (30.9)	100 (19.4)	242 (20.4)	127 (24.6)	276 (23.3)
**Years on DP before revascularisation**												
≤3	1556 (31.7)	4337 (40.9)	245 (15.7)	1060 (24.4)	186 (12.0)	454 (10.5)	652 (41.9)	1408 (32.5)	351 (22.6)	955 (22.0)	122 (7.8)	460 (10.6)
4–10	1975 (40.3)	4233 (40.0)	195 (9.9)	634 (15.0)	147 (7.4)	356 (8.4)	673 (34.1)	1104 (26.1)	327 (16.6)	694 (16.4)	633 (32.1)	1445 (34.1)
11–35	1372 (28.0)	2024 (19.1)	62 (4.5)	102 (5.0)	64 (4.7)	137 (6.8)	243 (17.7)	251 (12.4)	146 (10.6)	160 (7.9)	857 (62.5)	1374 (67.9)

^1^DP-diagnoses according to the International Classification of Diseases (ICD-10)

^2^gender-specific column percent of all with DP at time of intervention

^3^gender-specific row percent of all with DP at intervention in the different sub-groups

^4^Acute Coronary Syndrome: Non-ST-elevated or ST-elevated myocardial infarction.

More women (38%) than men (20%) and more CABG patients (26%) than PCI patients (22%) had DP at the time of revascularisation (Table 3). After adjustments for age and level of education, the OR for DP in women compared to men was 2.40 (95% CI: 2.29–2.50). After adjustments for gender, the OR for DP in CABG compared to PCI patients was 1.30 (95% CI: 1.26–1.35). Thus, both gender and type of intervention were of importance for DP, why also the logistic analyses were stratified by gender and type of intervention.

**Table 3:** Disability pension (DP) at the time of first coronary artery bypass graft surgery (CABG) or percutaneous coronary intervention (PCI), frequencies (n), proportions (%), crude and adjusted odds ratio (OR) with 95% confidence intervals (CI), by gender and type of intervention.

	**All**	**DP**	**Crude model**	**Model I**	**Model II**	**Model III**
	**n (%)**	**n (%)**	**OR (95% CI)**	**OR (95% CI)**	**OR (95% CI)**	**OR (95% CI)**
**Gender**						
Men	52,621 (80.1)	10,594 (20.1)	1	1	1	
Women	13,055 (19.9)	4,903 (37.6)	2.39(2.29–2.49)	2.38 (2.28–2.48)	2.40 (2.29–2.50)	
**Type of intervention**						
PCI	42,717 (65.0)	9,539 (22.3)	1	1	1	1
CABG	22,959 (35.0)	5,958 (26.0)	1.22 (1.17–1.27)	1.12 (1.08–1.16)	1.09 (1.05–1.13)	1.30 (1.26–1.35)
**All**	65,676 (100)	15,497 (23.6)				

Model I = adjusted for age; Model II = adjusted for age and level of education; Model III = adjusted for gender.

After multivariable adjustments and regardless of gender and type of intervention, the odds for DP at the time of revascularisation was higher among patients aged ≥50 years compared to those aged 30–49 years; especially in men, 60–63 years, with CABG (OR: 4.91; 95% CI: 4.27–5.66) (Table 4 and 5, model IV). Also, patients with elementary or high school compared to college/university education, had a higher OR for DP; especially men with PCI and elementary school (OR: 2.96; 95% CI: 2.69–3.26). A higher OR was found among foreign-born patients compared to those born in Sweden; especially men with PCI (OR: 2.11; 95% CI: 1.96–2.27) and among women with an indication of other diagnoses than ACS or stable angina pectoris for PCI (OR: 1.72; 95% CI: 1.31–2.24). On the contrary, the OR was lower among men who had their first revascularisation after 1996; lowest in men with CABG in year 2001–2003 (OR: 0.72; 95% CI: 0.63–0.81). There was no significant association between diabetes mellitus and DP at the time of revascularisation.

**Table 4:** Crude and adjusted odds ratio (OR) with 95% confidence interval (CI) for disability pension (DP) at time of a first coronary artery bypass graft surgery (CABG), among women and men, with regard to sociodemographic and medical factors.

	**Women (n = 1500)**			**Men (n = 4458)**		
	***Crude***	***Model I***	***Model IV***	***Crude***	***Model I***	***Model IV***
	**OR (95% CI)**	**OR (95% CI)**	**OR (95% CI)**	**OR (95% CI)**	**OR (95% CI)**	**OR (95% CI)**
**Age at intervention (years)**						
30–49	1	1	1	1	1	1
50–54	1.51 (1.16–1.95)		1.54 (1.18–2.02)	1.49 (1.28–1.73)		1.54 (1.32–1.81)
55–59	2.13 (1.69–2.68)		2.19 (1.72–2.80)	2.28 (1.99–2.62)		2.47 (2.14–2.85)
60–63	3.23 (2.57–4.05)		3.14 (2.47–4.01)	4.46 (3.89–5.10)		4.91 (4.27–5.66)
**Level of education**						
College or university	1	1	1	1	1	1
High school	1.63 (1.30–2.05)	1.70 (1.35–2.14)	1.75 (1.37–2.22)	1.93 (1.71–2.16)	1.98 (1.76–2.23)	1.95 (1.73–2.20)
Elementary school	2.91 (2.32–3.65)	2.70 (2.15–3.39)	2.79 (2.20–3.55)	2.86 (2.56–3.21)	2.70 (2.40–3.03)	2.63 (2.33–2.97)
**Country of birth**						
Other (ref: Sweden)	1.45 (1.23–1.72)	1.40 (1.18–1.66)	1.50 (1.25–1.81)	1.67 (1.54–1.82)	1.88 (1.73–2.05)	2.07 (1.89–2.26)
**Type of living area (cities and communities)**						
Larger	1	1	1	1	1	1
Medium	1.23 (1.04–1.45)	1.21 (1.02–1.44)	1.22 (1.01–1.46)	1.09 (1.00–1.19)	1.06 (0.98–1.16)	1.12 (1.02–1.22)
Smaller	1.45 (1.22–1.71)	1.41 (1.19–1.67)	1.48 (1.24–1.78)	1.21 (1.11–1.32)	1.17 (1.08–1.28)	1.28 (1.16–1.40)
**Year of intervention**						
1994–1996	1	1	1	1	1	1
1997–2000	0.89 (0.75–1.05)	0.89 (0.75–1.05)	0.89 (0.72–1.11)	0.87 (0.80–0.94)	0.85 (0.78–0.93)	0.90 (0.80–1.00)
2001–2003	0.92 (0.76–1.11)	0.92 (0.76–1.12)	0.94 (0.74–1.19)	0.73 (0.66–0.80)	0.69 (0.63–0.77)	0.72 (0.63–0.81)
2004–2006	1.02 (0.83–1.26)	0.98 (0.79–1.21)	1.03 (0.80–1.33)	0.80 (0.72–0.89)	0.72 (0.65–0.80)	0.75 (0.66–0.85)
**Indication for intervention**						
Stable angina pectoris	1	1	1	1	1	1
ACS^1^	1.12 (0.97–1.28)	1.14 (0.99–1.31)	1.13 (0.96–1.34)	1.20 (1.12–1.28)	1.23 (1.15–1.32)	1.16 (1.06–1.27)
Other	1.14 (0.80–1.62)	1.08 (0.79–1.47)	1.05 (0.75–1.48)	1.37 (1.14–1.64)	1.21 (1.03–1.41)	1.16 (0.98–1.37)
**Diabetes mellitus** (ref: no)	1	1	1	1	1	1
Yes	0.92 (0.75–1.14)	0.95 (0.76–1.18)	1.06 (0.82–1.36)	0.79 (0.72–0.88)	0.81 (0.73–0.89)	1.02 (0.90–1.15)
Missing	1.87 (1.49–2.34)	2.03 (1.61–2.55)	2.30 (1.76–2.99)	1.58 (1.42–1.76)	1.64 (1.46–1.83)	2.03 (1.78–2.31)

Model I = adjusted for age. Model IV = adjusted for all variables included.

**^1^** Acute Coronary Syndrome: Non-ST-elevated or ST-elevated myocardial infarction.

**Table 5:** Crude and adjusted odds ratio (OR) with 95% confidence intervals (CI) for disability pension (DP) at time of a first percutaneous coronary intervention (PCI), among women and men, with regard to socio-demographic and medical factors.

	**Women (n = 3403)**			**Men (n = 6136)**		
	*Crude*	*Model I*	*Model IV*	*Crude*	*Model I*	*Model IV*
**Age at intervention (years)**						
30–49	1	1	1	1	1	1
50–54	1.35 (1.16–1.59)		1.41 (1.21–1.65)	1.44 (1.30–1.59)		1.54 (1.38–1.72)
55–59	2.03 (1.77–2.32)		2.16 (1.87–2.49)	2.19 (1.99–2.40)		2.41 (2.19–2.65)
60–63	3.27 (2.86–3.75)		3.44 (2.98–3.96)	3.79 (3.46–4.16)		4.17 (3.78–4.60)
**Level of education**						
College or university	1	1	1	1	1	1
High school	1.85 (1.61–2.12)	1.92 (1.67–2.21)	1.87 (1.62–2.15)	2.08 (1.89–2.28)	2.17 (1.98–2.39)	2.20 (2.00–2.42)
Elementary school	2.82 (2.45–3.24)	2.68 (2.32–3.09)	2.60 (2.24–3.01)	3.08 (2.81–3.38)	2.96 (2.70–3.26)	2.96 (2.69–3.26)
**Country of birth**						
Other (ref: Sweden)	1.51 (1.36–1.69)	1.57 (1.40–1.75)	1.67 (1.48–1.88)	1.76 (1.65–1.88)	2.04 (1.91–2.18)	2.11 (1.96–2.27)
**Type of living area (cities and communities)**						
Larger	1	1	1	1	1	1
Medium	1.22 (1.10–1.35)	1.21 (1.09–1.34)	1.24 (1.11–1.39)	0.97 (0.91–1.04)	0.95 (0.89–1.02)	1.02 (0.95–1.10)
Smaller	1.21 (1.09–1.35)	1.19 (1.07–1.33)	1.24 (1.11–1.40)	1.04 (0.96–1.11)	1.00 (0.93–1.08)	1.08 (1.00–1.17)
**Year of intervention**						
1994–1996	1	1	1	1	1	1
1997–2000	0.93 (0.81–1.08)	0.93 (0.80–1.09)	0.87 (0.72–1.04)	0.86 (0.78–0.95)	0.82 (0.75–0.91)	0.84 (0.75–0.94)
2001–2003	1.00 (0.87–1.16)	0.96 (0.83–1.11)	0.85 (0.71–1.03)	0.83 (0.75–0.90)	0.75 (0.68–0.82)	0.75 (0.66–0.84)
2004–2006	1.25 (1.09–1.44)	1.19 (1.04–1.38)	1.11 (0.92–1.34)	0.90 (0.83–0.98)	0.78 (0.72–0.86)	0.81 (0.72–0.91)
**Indication for intervention**						
Stable angina pectoris	1	1	1	1	1	1
ACS^1^	0.97 (0.89–1.06)	1.00 (0.91–1.10)	1.00 (0.90–1.10)	0.95 (0.89–1.01)	0.96 (0.90–1.02)	1.00 (0.93–1.06)
Other	1.40 (1.09–1.79)	1.58 (1.23–2.04)	1.72 (1.31–2.24)	1.08 (0.92–1.26)	1.09 (0.93–1.28)	1.17 (0.99–1.38)
**Diabetes mellitus** (ref: no)						
Yes	1.12 (0.97–1.29)	1.07 (0.92–1.24)	1.11 (0.91–1.35)	0.84 (0.77–0.92)	0.79 (0.72–0.87)	0.94 (0.83–1.06)
Missing	2.30 (1.96–2.71)	2.30 (1.95–2.72)	2.37 (1.93–2.92)	1.69 (1.53–1.88)	1.54 (1.39–1.71)	1.74 (1.52–1.98)

Model I = adjusted for age. Model IV = adjusted for all variables.

^1^ Acute Coronary Syndrome: Non-ST-elevated or ST-elevated myocardial infarction.

## Discussion

In this nationwide study we investigated the prevalence of all-cause and diagnosis-specific DP at the time of CABG or PCI within the year 1994–2006, accounting for socio-demographic and medical factors. We found that 24% of the patients had DP, usually for full-time. This means that about a quarter already had left working life at the time of revascularisation. Moreover, in the studied population, the proportion of being on DP in 2004 was almost twice as large (22%) as in the general population aged 30–63 (12%) in 2004 (the year with the, so far, highest DP rate in Sweden) [38]. In the whole working population of OECD countries, the corresponding rates were 6% in 2007, 5% in 1990[39] and in Sweden 9% in 2007 [38].

Although the indication for intervention was cardiac symptoms, musculoskeletal diagnoses was the largest DP-diagnostic group; also in most of the subpopulations. This is also the largest DP-diagnostic group in the general population [9,40–42]. CVD was the second largest DP-diagnostic group (22%), however, the rate of DP due to CVD was much lower in the general population (6%) of Sweden in 2002 [42]. In the general population, mental diagnoses usually was the second largest DP-diagnostic group, e.g. 30% of all DPs in 2002 [42]. However, in this study mental diagnoses was only the third largest group (13%), also in most of the subpopulations. Of all with DP, 62% had had DP for at least four years before revascularisation. Thus, long-term DP (marginalisation from the labour market) in itself might be a risk factor for future CVD, something that requires other studies. There is hardly any scientific knowledge on consequences of being on DP. Some recent studies indicate that DP might be a risk indicator for mortality [43–48], also due to non-lethal DP diagnoses such as musculoskeletal diagnoses.

Regardless of gender, type of intervention or adjustments, and in accordance with previous studies of DP in the general population [17,23–33], we found a higher OR for DP among older patients, among lower level educated; especially men with PCI who almost had a tripled OR for DP, and among those born outside Sweden; especially men with PCI who had a doubled OR for DP at the time of revascularisation.

In line with previous studies of DP [12–22], the studied women were twice as likely as the men to have DP, also after adjustments for age and educational level. This indicates that also other factors were associated with the higher odds for DP in women. There are several theories on reasons for the higher DP rates in women [49,50]. Possible explanations regarding this patient group are women’s higher age at the diagnosis of cardiovascular disease [51] and that women who undergo CABG have more co-morbidity and smaller coronary arteries than men [2]. Moreover, women’s higher sick-leave rate [52], different work demands, and possible gender bias in health care [49,50] could be contributing factors to the higher DP rate in women.

Patients above 50 years of age had, as expected, a higher odds for DP; men, 60–63 years of age, had more than a fourfold OR for DP; this is only slightly above that of men in the general population of that age (30 vs 27%) [38].

This is the first study of DP prevalence at the time of first coronary revascularisation among working-aged women and men. In line with previous studies on DP in the general population [30–33], foreign-born individuals had a higher DP-rate than those born in Sweden, even after adjustments for other socio-demographic factors. However, in order to gain more knowledge on these associations, further specific studies are needed.

Diabetes mellitus was not associated with a higher OR for DP at time of revascularisation. Nevertheless, it is a risk indicator for future DP and long-term sick leave after coronary revascularisation [53,54]. This finding could be of importance in the rehabilitation of patients with diabetes mellitus at time of coronary revascularisation.

### Strengths and limitations

This is the first nationwide, population-based study that investigates the prevalence of all-cause and diagnosis-specific DP at the time of coronary revascularisation among working aged women and men. The main strengths are the large study population, that all patients, aged 30–63 years, with no on old-age pension, and with a first coronary revascularisation in Sweden between 1994–2006, were included, and the high quality of data linked from several registers [55]. Although most studies on DP do not have access to information on DP diagnoses at all, limitations are the non-available information on DP-diagnoses for DPs granted before 1994 and the missing data on diabetes mellitus. Diabetes data was collected by the physicians asking the patients a question at the time of the intervention. The reason for this missing data is unclear, but a likely explanation is that physicians did not register information if the patient did not have diabetes.

## Conclusions

About a quarter of patients aged 30–63 years already had DP at the time of their first coronary revascularisation, most often due to musculoskeletal DP diagnoses. More than half had had DP for at least four years before intervention. DP was associated with female gender, older age, lower educational level, and being born outside Sweden.
